# Investigation of a fluorescent reporter microenvironment niche labeling strategy in experimental brain metastasis

**DOI:** 10.1016/j.isci.2024.110284

**Published:** 2024-06-15

**Authors:** Matteo Massara, Bastien Dolfi, Vladimir Wischnewski, Emma Nolan, Werner Held, Ilaria Malanchi, Johanna A. Joyce

**Affiliations:** 1 Department of Oncology, University of Lausanne, 1011 Lausanne, Switzerland; 2 Ludwig Institute for Cancer Research, University of Lausanne 1011 Lausanne, Switzerland; 3 Agora Cancer Research Centre Lausanne, 1011 Lausanne, Switzerland; 4 L. Lundin and Family Brain Tumor Research Center, Departments of Oncology and Clinical Neurosciences, Centre Hospitalier Universitaire Vaudois, 1011 Lausanne, Switzerland; 5 Tumour-Host Interaction Laboratory, The Francis Crick Institute, London NW1 1AT, UK

**Keywords:** Microenvironment, Cell biology, Cancer

## Abstract

Brain metastases are the most common brain tumors in patients and are associated with poor prognosis. Investigating the colonization and outgrowth of brain metastases is challenging given the complexity of the organ, tissue sampling difficulty, and limited experimental models. To address this challenge, we employed a strategy to analyze the metastatic niche in established lesions, based on the release of a cell-penetrating mCherry tag from labeled tumor cells to neighboring niche cells, using different brain metastasis mouse models. We found that CD206+ macrophages were the most abundant cells taking up the mCherry label in established metastases. *In vitro* and *in vivo* experiments demonstrated that macrophages uptake and retain the canonical form of mCherry, even without the cell-penetrating portion of the tag. These results identify a specific macrophage subset in the brain that retains tumor-supplied fluorescent molecules, thereby complicating the long-term use of niche labeling strategies in established experimental brain metastasis.

## Highlights

• The sLP-mCherry strategy detected host cells communicating with BrM tumor cells
• CD206+ macrophages are associated with high uptake of sLP-mCherry
• mCherry is taken up by CD206+ macrophages, even without the cell-penetrating tag

## Graphical abstract

**Figure:**
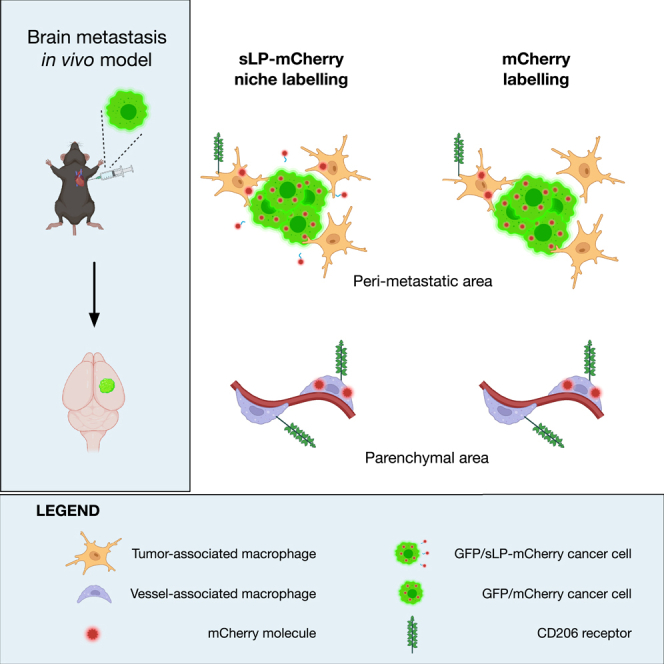

## Introduction

Metastasis to the brain is a common occurrence in patients, notably from lung (40%–60%), breast (15%–25%), or melanoma (5%–20%) primary cancers, and represents a major clinical challenge.^1^^,^^2^ There are currently no broadly effective therapies for brain metastasis (BrM), and patient survival is often measured by just a few months following a BrM diagnosis.^1^ As such, there is an urgent need for new perspectives to understand the mechanisms that govern BrM and consequently identify effective therapeutic strategies. One such approach is to investigate the communication between cancer cells and non-cancerous immune and stromal cells within the complex, multicellular tumor microenvironment (TME), which can result in tumor-promoting effects by the host during metastatic dissemination and outgrowth.^3^^,^^4^ Intercellular communication within the TME occurs through a variety of mechanisms including via direct cell contact (e.g., gap junction molecules, ligand-receptor interactions, and tunneling nanotubes) and paracrine means (e.g., extracellular vesicles, cytokines, growth factors, and metabolites).^4^

To investigate this complex communication, several tools have recently been developed to unravel the interactions between cancer and host cells *in situ* within the TME.^5^ One such strategy is an *in vivo* niche labeling system, whereby cancer cells are transduced with a secreted lipid-permeable mCherry tag (termed sLP-mCherry), which is subsequently released by cancer cells and taken up by cells residing in proximity to the labeled cells.^6^^,^^7^ This technique was first used to study the early steps of metastatic colonization in experimental breast-to-lung metastasis models through an unbiased approach.^6^^,^^7^ This led to the identification of a subset of alveolar epithelial stem cells which were important for lung-metastatic niche formation.^6^ Other methods have similarly been utilized for *in vivo* labeling of neighboring cells, for example, through the incorporation of a Cre-dependent fluorophore switch in benign breast tumor cells, coupled with Cre protein exposure/secretion by malignant cells.^8^ This strategy was used to investigate phenotypic alterations mediated by cancer cells, including via extracellular vesicles.^8^

By comparison to other organs, the brain TME represents several unique cell types and singular properties, including the presence of the blood-brain barrier and several meningeal layers, which tightly regulate the entry of cells and substances into the brain.^3^ Previous studies have shown that the communication between cancer cells and host cells in BrM is complex and involves multiple cellular components^4^^,^^9^ including tumor-associated microglia and macrophages,^10^^,^^11^ neutrophils,^12^ astrocytes,^13^^,^^14^ the vasculature,^15^ and neurons.^16^^,^^17^ In this study, we utilized the niche labeling strategy outlined earlier^6^^,^^7^ in BrM models *in vivo*, to investigate the cellular composition and spatial localization of the niche compartment in established tumors.

## Results

### The mCherry niche labeling system in BrM

**Figure 1:**
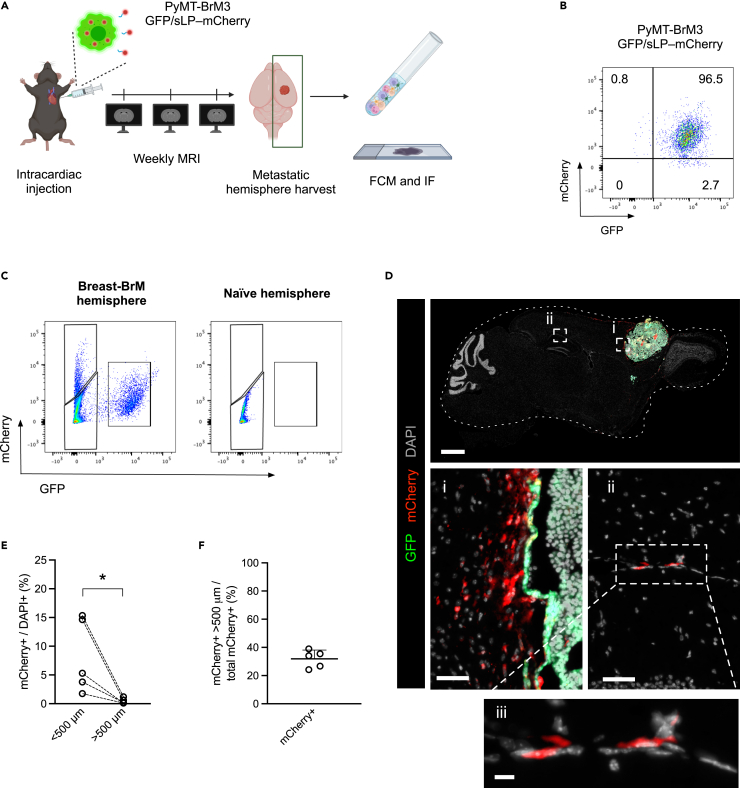
The mCherry niche labeling system in brain metastasis reveals mCherry+ cells outside the metastatic niche in established breast-BrM (A) Experimental schematic for investigation of the metastatic niche in breast-to-brain metastasis (BrM). Briefly, immunocompetent mice were injected intracardially with GFP/sLP-mCherry-transduced PyMT-BrM3 breast cancer cells and monitored by weekly MRI to assess BrM outgrowth. At the experimental endpoint, the brain hemispheres where the BrM was detected by MRI were collected by performing a sagittal cut and processed for flow cytometry (FCM) and immunofluorescence (IF) staining analyses. (B) Representative FCM plot of the GFP/sLP-mCherry-labeled PyMT-BrM3 cell line cultured *in vitro*. (C) Representative FCM plots of single-cell suspensions from the labeled PyMT-BrM3 breast-to-brain metastatic hemisphere (left) and the naive uninjected brain tissue (right) used as negative reference for mCherry expression in the FCM analysis. (D) Representative IF image of whole-brain tissue section staining. Scale bar 1 mm in top panel. i) Higher magnification of the peri-metastatic area, showing the GFP+ mCherry+ cancer cells on the right side of the image. Scale bar 50 μm. ii) Higher magnification of a parenchymal area considerably further than 500 μm from the tumoral lesion, showing GFP-neg mCherry+ cells. Scale bar 50 μm. iii) Higher magnification of the parenchymal area (ii) shown above. Scale bar 10 μm. (E) Quantification of mCherry+ DAPI+ cells as a proportion of total DAPI+ cells stratified by the distance between the positive cells and the tumor lesion (<500 μm or >500 μm) using QuPath. Dashed lines indicate paired samples. Metastatic hemispheres from *n* = 5 mice. (F) Percentage of the number of mCherry+ DAPI+ cells detected at a distance from the tumor lesion (>500 μm) compared to the total mCherry+ DAPI+ cell count as detected in the whole-brain tissue slices. Metastatic hemispheres from *n* = 5 mice. Statistical analysis in (E) was performed using paired t test. Data are represented as mean ± SD. ∗, *p* < 0.05.

To explore tumor-to-host communication in BrM, we used a labeling strategy that involved the transduction of cancer cells with a plasmid encoding both green fluorescent protein (GFP) and a modified mCherry protein containing a signal peptide (for secretion) followed by a lipid-permeable transactivator of transcription (TAT) domain (sLP-mCherry) (Figures 1A and S1A). We applied this approach to label experimental breast- and lung-BrM cell lines (PyMT-BrM3 and LLC-BrM3, respectively), which were then used to generate *in vivo* BrM mouse models. Using this labeling system, the cancer cells both express GFP and express/secrete the sLP-mCherry fluorophore protein. Consequently, cells in their proximity can endocytose and store the secreted sLP-mCherry tag in multi-lamellar bodies, as previously described.^6^^,^^7^ We found that transduced brain-homing cancer cells show a similar expression pattern *in vitro* for both breast-BrM (Figure 1B) and lung-BrM (Figure S1B) cell lines after being labeled with the GFP/sLP-mCherry construct. Following *in vivo* injection and metastasis engraftment, we could discriminate the labeled cells by flow cytometry (FCM, Figures 1C and S1C), enabling the separation of cancer cells (GFP+ mCherry+) from metastasis-associated niche cells (GFP-neg mCherry+). Normal cells distant from the metastatic lesion should not endocytose the sLP-mCherry tag and thus will not be labeled (GFP-neg mCherry-neg). In both BrM models, only a fraction of cells in the metastasis-containing brain tissue were attributed to the niche compartment (Figures S1D and S1E), which is consistent with the original studies using the 4T1 breast-to-lung metastasis model.^6^^,^^7^

Interestingly, by imaging entire tissue sections of the brain, we were able to detect the presence of sLP-mCherry+ cells both proximal to, and at a distance from, the metastatic lesion, using immunofluorescence (IF) staining and image analysis in the breast-BrM model (Figure 1D). As expected, we found sLP-mCherry+ cells in proximity to the tumoral mass (Figure 1D, panel i and S1F). However, we also detected mCherry+ cells at a considerable distance from the metastatic lesion (Figure 1D, panel ii). Importantly, for IF analyses we included only mice with a solitary BrM by magnetic resonance imaging (MRI) (Figure S1G), to exclude the potential complication of other BrM lesions in different 3D planes interfering with the signal. We then performed IF image analyses on each slide, defining the peritumoral area at an arbitrary setting of 500 μm from the border of the tumoral mass. This distance was defined based on being 10x the average distance of sLP-mCherry uptake by metastatic niche cells in the breast-to-lung model, as reported in the original publication.^6^ While the relative abundance of sLP-mCherry+ cells outside of the metastatic niche was lower (Figure 1E), we nonetheless detected a substantial amount of sLP-mCherry+ cells in the distal-tumoral area proportionate to the total sLP-mCherry fraction (Figure 1F). Moreover, sLP-mCherry staining by IF revealed a uniform cell labeling pattern in the distal-tumoral area (Figure 1D, panel iii). This pattern of staining contrasts with the original study investigating breast-to-lung metastasis, in which the lung niche cells displayed retention of the sLP-mCherry protein within multi-lamellar bodies.^6^^,^^7^ Together, our results indicate a distinct labeling in established breast-BrM lesions compared to the breast-to-lung metastasis intravenous seeding model previously described,^6^^,^^7^ in which only those cells in direct proximity to the lung-metastatic lesion were reported as being labeled at the early stages of metastasis.

### Tumor-associated macrophages are the most abundant cell type detected using the GFP/sLP-mCherry labeling strategy

**Figure 2:**
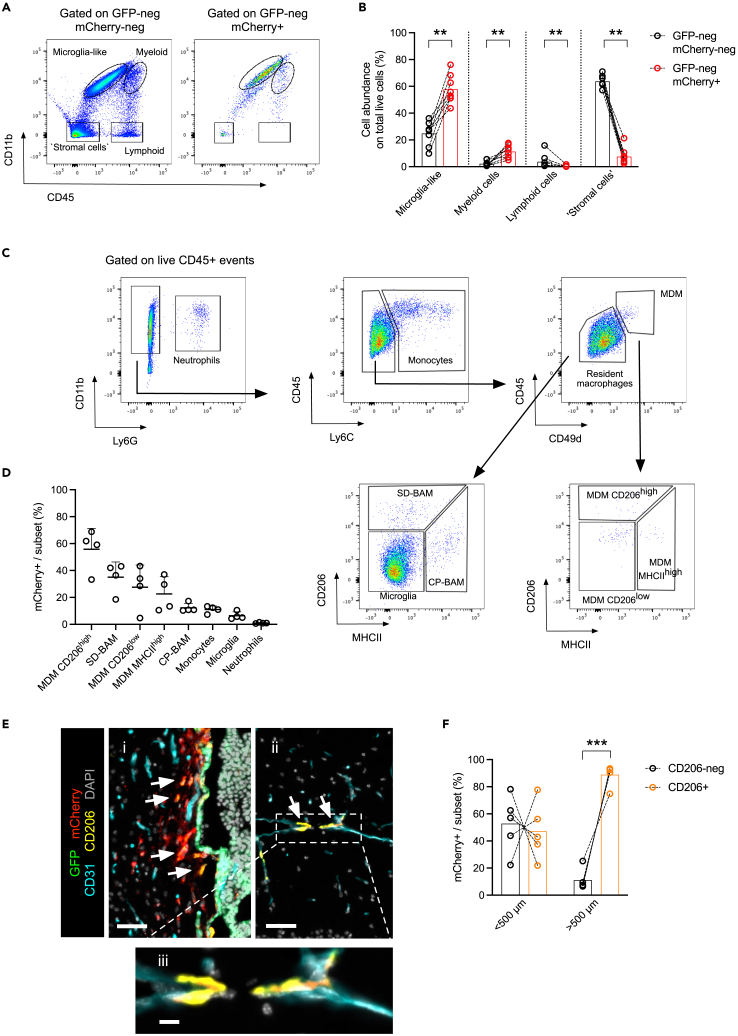
CD206+ macrophages are the most abundant cell population within the sLP-mCherry+ fraction and are located outside of the metastatic niche in the breast-BrM model (A) Representative flow cytometry plots of the GFP-neg mCherry-neg and GFP-neg mCherry+ cell fractions in the breast-BrM model, showing the gating strategy used. Microglia-like cells were defined as CD45^low^ CD11b^high^, myeloid cells as CD45^high^ CD11b^high^, lymphoid cells as CD45^high^ CD11b-neg, and “stromal cells” as CD45-neg CD11b-neg. (B) Relative quantification of cell populations in the GFP-neg mCherry-neg and in the GFP-neg mCherry+ fractions in the breast-BrM metastasis model. Dashed lines indicate paired samples. Metastatic hemispheres from *n* = 8 mice. (C) Representative FCM gating strategy to identify neutrophils and the different macrophage subpopulations. Neutrophils were identified as CD11b+ Ly6G + events, monocytes as Ly6C+ CD11b+ Ly6G-neg events, and monocyte-derived macrophages (MDMs) as CD45^+^ CD49d+. Among CD49d-neg events, subdural border-associated macrophages (SD-BAMs) were identified as CD206+ cells, choroid plexus border-associated macrophages (CP-BAMs) as MHCII+, and microglia were identified as CD206-neg MHCII-neg events. Among the CD49d+ events, three subsets of MDMs were detected based on CD206 and MHCII expression. (D) Quantification of relative mCherry uptake by the different cell populations in the metastatic hemisphere in the breast-to-brain metastasis model as a percentage of the total number of cells in each indicated subset. Metastatic hemispheres from *n* = 4 mice. (E) Representative IF images of brain tissue in the breast-BrM model. i) The peri-metastatic area. Scale bar 50 μm. ii) A parenchymal area of brain tissue, considerably further than 500 μm from the tumoral lesion. Scale bar 50 μm. iii) Detail of the parenchymal area shown above in (ii). Scale bar 10 μm. White arrows in (i) and (ii) indicate mCherry+ CD206+ cells. (F) Relative quantification by IF image analysis of mCherry+ DAPI+ cells in the CD206+ and CD206-neg fractions, stratified based on the distance between the detected cells and the tumoral lesion. Dashed lines indicate paired samples. Metastatic hemispheres from *n* = 5 mice. Statistical analysis in (B) was performed using paired t test with Wilcoxon correction. Statistical analysis in (F) was performed using paired t test. Data are represented as mean ± SD. ∗∗, *p* < 0.01; ∗∗∗, *p* < 0.001.

To investigate which cell type was predominantly stained by the sLP-mCherry tag at a considerable distance from the tumoral mass, we performed FCM analysis of the metastatic brain hemisphere tissues (Figures 1A and S1A). We evaluated CD45 and CD11b expression in single-cell suspensions (Figures 2A and S2A), comparing both GFP-negative (neg) mCherry-neg cells and GFP-neg mCherry+ cells (Figures 1C and S1C). We detected microglia-like cells (CD45^low^ CD11b^high^), myeloid cells (CD45^high^ CD11b^high^), lymphoid cells (CD45^high^ CD11b-neg), and “stromal cells” (CD45-neg CD11b-neg) (Figures 2A and S2A). By comparing both GFP-neg mCherry-neg cells and GFP-neg mCherry+ cells (Figure 1C) in the breast-BrM model, a relative increase of sLP-mCherry uptake by microglia-like and myeloid cells was evident in the niche compartment (Figure 2B), based on total viable cells. By contrast, stromal and lymphoid cells in the niche showed a higher proportion of GFP-neg mCherry-neg versus GFP-neg mCherry+ cells. We made a similar finding in the lung-BrM model (Figure S2B). FCM analysis of brains from healthy non-tumor-bearing mice shows the physiological proportions of these cell populations in the normal brain (Figure S2C). Thus, we conclude that tumor-associated macrophages (TAMs) are the most abundant mCherry-labeled cell type in the brain metastatic niche.

### CD206 is associated with the uptake of sLP-mCherry outside the brain metastatic niche

To determine which specific TAM subpopulations were present in the niche fraction, we assessed sLP-mCherry uptake in different myeloid cell subsets by FCM. Using a gating strategy (Figure 2C), including cell-surface markers previously reported to discriminate different macrophage populations^18^^,^^19^ and using the naive hemisphere as negative reference for the mCherry expression in each population (Figure S2D), we detected the highest sLP-mCherry abundance in macrophage subsets characterized by CD206 expression in both the breast-BrM and lung-BrM models (Figures 2D and S2E). While CD206 positivity was associated with a higher relative mCherry uptake, this macrophage subset represented a minor population within the total immune cell compartment, with microglia and monocytes representing the most abundant immune cells overall in both breast- and lung-metastatic brain tissues (Figures 2F and S2G). Together, these data show that CD206 is a cell-surface marker associated with high sLP-mCherry uptake in metastatic brain tissue. To assess the spatial organization of CD206+ cells, we performed IF staining and analysis in whole-tissue sections from the metastatic brain in the breast-BrM model. We observed an overlap between the mCherry and CD206 staining patterns in host cells (Figure 2E, panels i, ii). We then stratified these data based on the distance from the tumor mass, which revealed a prevalence of mCherry+ DAPI+ CD206+ cells outside the metastatic niche compared to mCherry+ DAPI+ CD206-neg cells (Figure 2F). The mCherry+ DAPI+ CD206+ cells located outside the metastatic niche were found in proximity to the CD31^+^ vasculature (Figure 2E, panels ii, iii), indicating that these cells may be perivascular macrophages. Finally, we observed a gradient of staining intensity for the sLP-mCherry+CD206+ cells (Figure S2H), with an accumulation of labeled CD206+ cells closer to the tumoral mass. In sum, these data show that vessel-associated CD206+ host cells, positive for the sLP-mCherry tag, can be found at considerable distances from the brain metastatic local niche.

### CD206 expression by macrophages is correlated with mCherry retention *in vitro*

**Figure 3:**
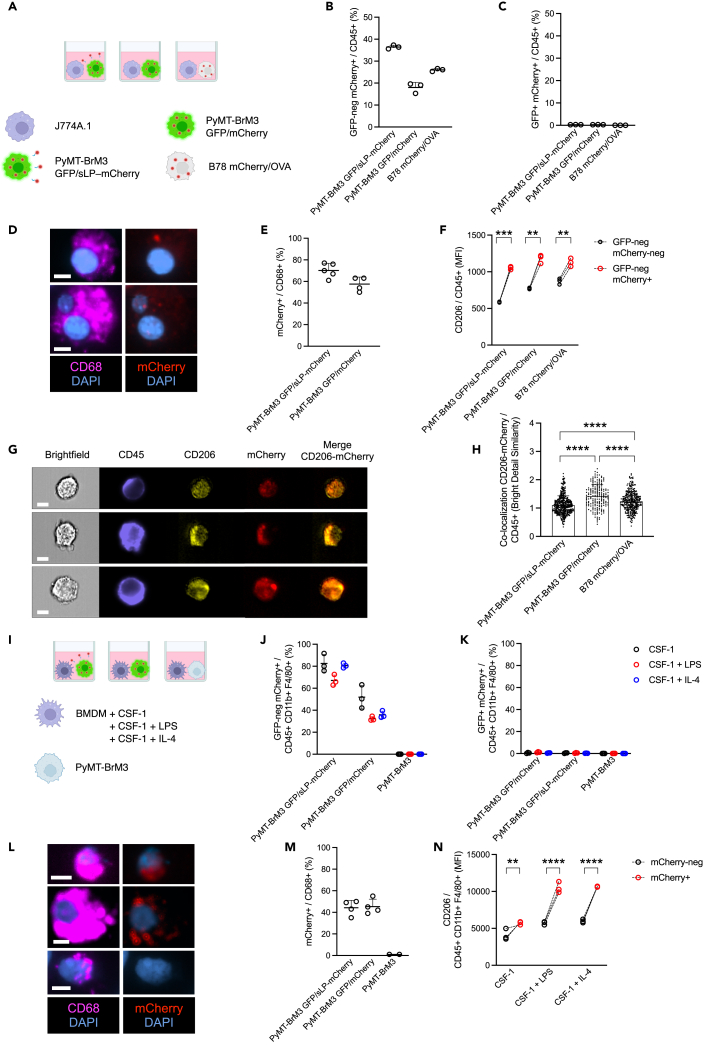
mCherry is retained by macrophages in cell co-cultures and is associated with CD206 expression (A) Schematic of the experimental design and the cell lines used for co-culture analyses involving J774A.1 as macrophage cell line. (B and C) Relative quantification of (B) GFP-neg mCherry+ and (C) GFP+ mCherry+ expression associated with J774A.1 macrophages, detected as CD45^+^ cells by FCM. (D) Representative cytospin IF images from the co-culture experiment. Top panel: J774A.1 macrophages (CD68^+^) co-cultured with PyMT-BrM3-GFP/sLP-mCherry cell line, bottom panel J774A.1 co-cultured with PyMT-BrM3-GFP/mCherry cell line. Scale bar 7 μm. (E) Relative quantification of mCherry+ expression associated with J774A.1 macrophages detected as CD68^+^ cells by IF staining. (F) CD206 levels as quantified by mean fluorescence intensity (MFI) in J774A.1 CD45^+^ macrophages, either GFP-neg mCherry-neg or GFP-neg mCherry+. Dashed lines indicate paired samples. (G) Representative images of the imaging flow cytometry experiment. Top panel: J774A.1 macrophages co-cultured with PyMT-BrM3-GFP/sLP-mCherry cell line, middle panel J774A.1 co-cultured with PyMT-BrM3-GFP/mCherry cell line, bottom panel J774A.1 co-cultured with B78 mCherry/OVA cell line. Scale bar 7 μm. (H) Quantification of CD206-mCherry co-localization in macrophages by imaging flow cytometry. *n* = 435 cells counted for PyMT-BrM3-GFP/sLP-mCherry co-cultures, *n* = 192 for PyMT-BrM3-GFP/mCherry co-cultures, *n* = 350 for B78 mCherry/OVA co-cultures. (I) Schematic of the experimental design and the cell lines used for co-culture analyses with BMDMs as the macrophage source. (J and K) Relative quantification of (J) GFP-neg mCherry+ and (K) GFP+ mCherry+ expression associated with BMDMs, detected as CD45^+^ CD11b+ F4/80+ cells by FCM. (L) Representative cytospin IF images from the co-culture experiment. Top panel: BMDMs (CD68^+^) co-cultured with PyMT-BrM3-GFP/sLP-mCherry cell line, middle panel BMDMs co-cultured with PyMT-BrM3-GFP/mCherry cell line, bottom panel BMDMs co-cultured with PyMT-BrM3 cell line. Scale bar 7 μm. (M) Relative quantification of mCherry+ expression associated with BMDMs detected as CD68^+^ cells by IF. (N) CD206 MFI of CD45^+^ CD11b+ F4/80+ BMDMs co-cultured with PyMT-BrM3 GFP/mCherry cells, either GFP-neg mCherry-neg or GFP-neg mCherry+. Dashed lines indicate paired samples. In (B, C, and F), *n* = 3, experiment repeated 3 independent times, one representative experiment is shown. (E), *n* = 5 for PyMT-BrM3 GFP/sLP-mCherry and *n* = 4 for PyMT-BrM3 GFP/mCherry, sum of two independent experiments. (H), single experiment. In (J, K, and N), *n* = 3, experiment repeated 2 independent times, one representative experiment is shown. (M), *n* = 4 for PyMT-BrM3 GFP/sLP-mCherry and PyMT-BrM3 GFP/mCherry. *n* = 2 for PyMT-BRM3, single experiment. Statistical analysis in (F and N) was performed using two-way ANOVA. Statistical analysis in (H) was performed using one-way ANOVA. Data are represented as mean ± SD. ∗∗, *p* < 0.01; ∗∗∗, *p* < 0.001; ∗∗∗∗, *p* < 0.0001.

To investigate the potential mechanism of sLP-mCherry uptake outside the local metastatic niche, we further analyzed the labeling system *in vitro* (Figure 3A). We co-cultured a murine macrophage cell line (J774A.1) with the PyMT-BrM3 cancer cell line that was transduced with either the sLP-mCherry construct (Figure 1B) or a mCherry version without the sLP portion (Figure S3A), which is non-secreted and non-cell permeant. In addition, co-culture with the melanoma cell line B78 mCherry/ovalbumin (OVA) was used as another control (Figure S3B), to be independent of tumor type. Unexpectedly, after 72 h of co-culture, we detected a considerable fraction of macrophages (CD45^+^ cells, Figure S3C) retaining mCherry (detected as GFP-neg mCherry+ events by FCM) in the co-culture condition with cancer cell lines transduced with the non-cell permeant mCherry version (i.e., lacking the sLP; Figures 3B and S3D). By contrast, GFP+ mCherry+ events were barely detectable in these macrophages (Figures 3C and S3D), indicating the retention of the mCherry protein by macrophages. This result is consistent with the GFP protein being quenched in the acidic environment of the lysosomes, as reported in multiple studies in mammalian cells and in bacteria while the mCherry protein is known to be stable under these conditions.^20^^,^^21^^,^^22^

We further validated the presence of mCherry in J774A.1 macrophages by performing IF staining of the co-cultures at the endpoint (Figures 3D and 3E). We also assessed CD206 expression by FCM in the co-cultures and found higher levels of CD206 in CD45^+^ mCherry+ GFP-neg cells compared to the CD45^+^ mCherry-neg GFP-neg cells in the conditions we evaluated (Figure 3F). By FCM, we also detected increased side scatter (SSC-A) values in CD45^+^ mCherry+ GFP-neg cells (Figure S3E), suggesting a potential morphological alteration associated with mCherry uptake. We next assessed the overlap between CD206 and mCherry by imaging FCM (Figure 3G) and detected the highest proportion of co-localization in J774A.1 cells co-cultured with the PyMT-BrM3 GFP/mCherry cell line (Figure 3H). We then performed similar co-culture experiments but separated the cancer cells and macrophages via a 0.4 μm permeable membrane (Figure S3F). As expected, we observed sLP-mCherry uptake (detected as GFP-neg mCherry+ events) in macrophages co-cultured with PyMT-BrM3 GFP/sLP-mCherry cells (Figures S3G and S3I). However, there was no mCherry retention (detected as GFP-neg mCherry+ events) in macrophages with PyMT-BrM3 GFP/mCherry and B78 mCherry/OVA cells (Figures S3G and S3I). This result indicates that the uptake of mCherry protein in the absence of the sLP portion is via a non-soluble mechanism, and dependent on cell contact. Furthermore, in this setting, GFP+ mCherry+ events were not detected in macrophages under any of the experimental conditions (Figures S3H and S3I).

We also performed the direct co-culture experiment using murine primary bone marrow-derived macrophages (BMDMs) generated through *in vitro* administration of colony-stimulating factor (CSF)-1. Following differentiation, we added either LPS or interleukin (IL)-4 in the media of BMDMs to promote different macrophage activation states. BMDMs were then directly co-cultured with either the PyMT-BrM3 GFP/mCherry cells lacking the sLP construct or the PyMT-BrM3 GFP/sLP-mCherry cell line as a positive control, or the unlabeled PyMT-BrM3 cell line as a negative control (Figure 3I). Independently of macrophage activation status, we found mCherry uptake by BMDMs also without the sLP construct (Figure 3J), confirming our earlier data generated using J774A.1 cells. By contrast, we detected almost no GFP signal in BMDMs co-cultured with each of the different cancer cell lines (Figure 3K). We next validated mCherry presence in BMDMs by IF staining (Figures 3L and 3M). By FCM, we also detected higher levels of CD206 in CD45^+^ CD11b+ F4/80+ GFP-neg mCherry+ cells compared to the mCherry-neg cells among the different co-cultures we evaluated (Figure 3N). Finally, we stained cancer cells using a commercial cell tracker dye prior to the start of the co-culture to assess a different cancer cell labeling system. We found a similar uptake of this cell tracker dye by BMDMs, independently of the macrophage activation status and cancer cell line used (Figure S3J).

### Cancer-cell-derived mCherry, without the sLP portion, is also retained by host cells in breast-to-brain metastatic tissues

**Figure 4:**
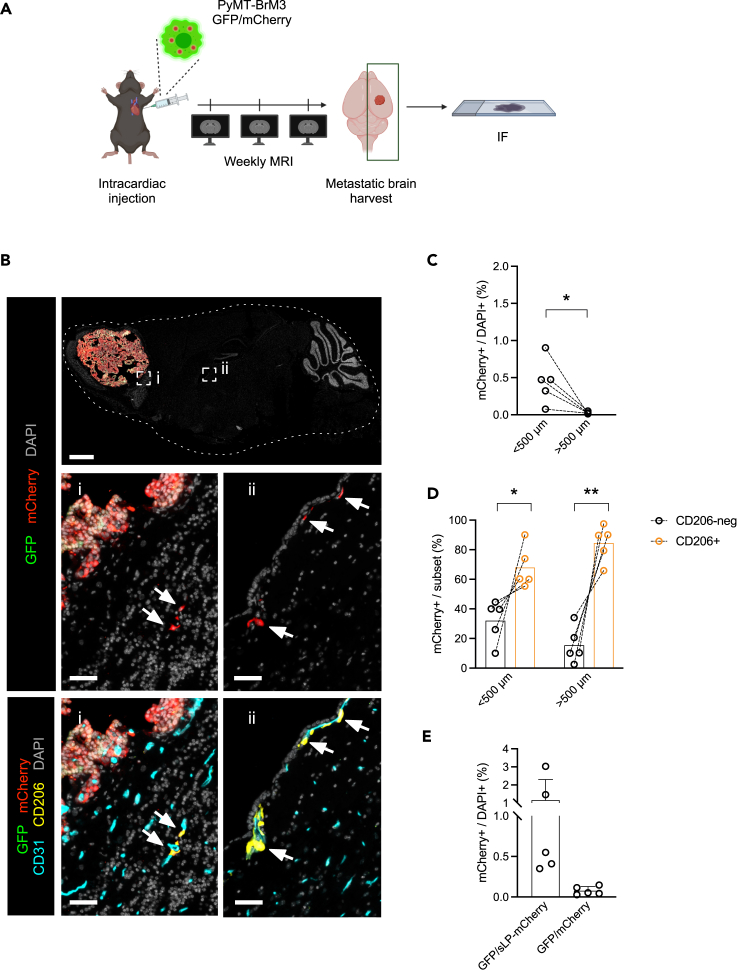
mCherry uptake by host cells in breast-BrM *in vivo*, in the absence of the cell-penetrating sequence (A) Schematic for the experimental investigation of the metastatic niche in breast-BrM. Briefly, immunocompetent mice were injected intracardially with GFP/mCherry-transduced PyMT-BrM3 cells (without the sLP portion) and monitored by weekly MRI to assess BrM outgrowth. At the experimental endpoint, the brain hemispheres where the BrM was detected by MRI were collected by performing a sagittal cut and processed for IF analysis. (B) Top panel: representative IF image of whole brain tissue section staining. Scale bar 1 mm. i) Higher magnification of the peri-metastatic area, with the GFP+ cancer cells on the top left of the image. Scale bar 50 μm. ii) Higher magnification of a parenchymal area considerably further than 500 μm from the tumoral lesion, showing GFP-neg mCherry+ cells. Scale bar 50 μm. White arrows indicate mCherry+ CD206+ cells, with CD206 and CD31 staining together shown in the lower panels. (C) Quantification of mCherry+ DAPI+ cells as a proportion of total DAPI+ cells stratified by the distance between the cells and the tumor lesion as calculated by QuPath. Dashed lines indicate paired samples. Metastatic hemispheres from *n* = 5 mice. (D) Relative quantification by IF image analysis of mCherry+ DAPI+ cells in the CD206-neg and CD206+ fractions, stratified based on the distance between the detected cells and the tumoral lesion. Dashed lines indicate paired samples. Metastatic hemispheres from *n* = 5 mice. (E) Relative quantification of mCherry+ DAPI+ cells as a proportion of total DAPI+ cells by IF image analysis. Metastatic hemispheres from *n* = 5 mice for both GFP/sLP-mCherry and GFP/mCherry constructs. Statistical analysis in (C and D) was performed using paired t test. Data are represented as mean ± SD. ∗, *p* < 0.05; ∗∗, *p* < 0.01.

To determine whether these cell culture findings were applicable *in vivo*, we injected the PyMT-BrM3 GFP/mCherry cell line (non-SLP variant) intracardially in mice to generate breast-BrM (Figure 4A). We analyzed the metastatic tissues by IF staining and detected mCherry+ cells in the brain parenchyma (Figure 4B panels i, ii). In line with the evidence of a contact-dependent mCherry labeling (Figure S3G), there was a higher frequency of mCherry+ cells in proximity to the metastatic lesion (Figure 4B, panel i) compared to mCherry+ cells distant to the metastatic niche (Figure 4B, panel ii) (quantified in Figure 4C). By MRI, we confirmed the presence of a solitary BrM in those brains (Figure S4A), and we determined by IF a similar ratio of mCherry+ cells in the brain located >500 μm from the metastatic lesion to the total proportions of mCherry cells in the aforementioned PyMT-BrM3 GFP/mCherry model and in the PyMT-BrM3 GFP/sLP-mCherry variant (Figures S4B and 1F). We also detected a gradient of mCherry+CD206+ cells with an accumulation of labeled CD206+ cells closer to the tumoral mass (Figure S4C), as with the sLP-mCherry construct (Figure S2H). In addition, we found mCherry+ CD206+ cells as a prevalent cell population both in proximity to and outside the metastatic niche (Figure 4D) adjacent to blood vessels (Figure 4B, lower panels). The mCherry labeling pattern with the non-sLP variant (Figure 4B, middle panels) resembled the labeling pattern (Figure 1D, panel iii) of the sLP-mCherry construct in metastatic brains. Finally, assessing the mCherry uptake by all cells using IF comparing the sLP-mCherry and mCherry variants, we found a higher labeling proportion using the sLP-mCherry construct (Figure 4E), as expected, thus indicating the overall functioning of niche labeling using the sLP-mCherry system. Collectively, our data indicate the ability of CD206+ macrophages in the brain TME to take up mCherry protein with high affinity, in a liposoluble tag (sLP)-independent manner, in established brain metastatic tumors, which can be found at a considerable distance from the labeled cancer cells.

## Discussion

Investigating the complex TME in primary and metastatic cancers has revealed various mechanisms of tumor-host interactions,^4^ and different methods have been developed in recent years to detect communication between cancer and host cells. One such approach involves labeling proximal TME niche cells, which are in communication with metastatic cancer cells, by first transducing cancer cells with a plasmid encoding both GFP and a cell-permeable (sLP) mCherry molecule.^6^ We used this niche labeling strategy herein with BrM models from either breast cancer or lung cancer origin. Our results revealed the presence of mCherry+ cells at considerable distances outside the metastatic niche compartment in established breast-BrM, which were found to be macrophages expressing CD206, and located in proximity to blood vessels. Moreover, based on our *in vitro* and *in vivo* data, the traditional form of mCherry (without the sLP portion) was also taken up by macrophages with high CD206 expression in a contact-dependent manner.

The GFP/sLP-mCherry niche labeling system has been used *in vivo* in several independent models, with a prominent proportion of mCherry+ macrophages evident in these different settings, including breast-to-lung^6^ and prostate-to-liver^23^ models. The cancer cell lines used in these other studies show a shorter metastatic latency and a faster outgrowth compared to the PyMT-BrM3 cells^24^ we have used herein, with the development of several metastases in the same organ. It is plausible that TAMs engulf metastatic cells since they can be recruited by cancer cells via different chemoattractant molecules and that this phenomenon would be more prevalent in labeled cancer cells which are growing for longer periods in the organ. In addition, as recently reported in a different context investigating antigen uptake using a melanoma cancer cell line labeled with a panel of fluorescent molecules, a variable percentage of labeled TAMs and dendritic cells in the draining lymph node was reported for each of the different fluorescent labels.^25^

Together, our data indicate a potential communication between cancer cells and vessel-associated CD206+ macrophages even at a considerable distance from established BrM. The mannose receptor, CD206, is a C-type lectin receptor that internalizes different endogenous and pathogen-associated ligands. CD206 is expressed mainly by myeloid cells and is associated with an M2-like polarization state,^26^^,^^27^ while it is constitutively expressed by border-associated macrophages in the brain.^19^ Future experiments will be important to investigate how macrophages are taking up the mCherry protein, the potential roles of these cells in the metastatic process, and their presence at a considerable distance from cancer cells. It will also be of interest to investigate the association between CD206 expression and sLP-Cherry and mCherry uptake and fluorescence retention in brain macrophages.

In sum, the investigation of the metastatic niche using the GFP/sLP-mCherry system revealed several unexpected challenges within the unique environment of long-term BrM. Moreover, these data suggest that, to avoid the unexpected effect of fluorescently labeled cells growing for extended periods of time in the tissue, the use of short-term inducible labeling systems could be explored. This study also underscores the importance of performing the relevant technical controls and the need to carefully interpret results regarding phagocytic cells such as macrophages in systems where fluorescent reporters are incorporated, both in cancer and in other pathological and physiological contexts.

### Limitations of the study

There are some limitations to our study. We evaluated metastasis-bearing brain tissue at the endpoint, which may result in potential differences compared to previous studies using this niche labeling system at earlier time points in different organs and mouse models. The PyMT-BrM3 breast-BrM model is an oligometastatic BrM model that phenocopies BrM development in patients. For IF analyses, we purposely selected mice with only a solitary BrM to assess mCherry distribution in the brain without the added complication of other BrM lesions in different 3D planes potentially interfering with the signal. It is possible that this specific inclusion criterion may lead to a source of potential bias in the representation of the model. Moreover, the presence of mCherry+ cells in the area outside the metastatic niche was evaluated in the experimental breast-BrM model alone. While other findings were confirmed using the lung-BrM model, analysis of the spatial distribution of macrophages was not performed for lung-BrM, as the labeled cancer cells are injected intracranially in this context, and thus could potentially spread within the meninges, which are enriched in CD206+ macrophages. In addition, further experiments are needed to investigate the mechanism of mCherry retention in perivascular macrophages in the area distant from the brain metastatic niche in relation to the co-culture results, which indicates a contact-dependent mechanism of mCherry uptake by macrophages. Collectively, investigating the role of resident CD206+ macrophages in BrM remains to be fully elucidated and represents an important scientific question to address.

## STAR★Methods

### Key resources table

**Table:** 

REAGENT or RESOURCE	SOURCE	IDENTIFIER
**Antibodies**		

FCM: CD45 AF700, rat monoclonal anti-mouse (clone 30-F11), dilution 1:200	Biolegend	Cat# 103128; RRID: AB_493715
FCM: CD45 BUV661, rat monoclonal anti-mouse (clone 30-F11), dilution 1:500	BD Biosciences	Cat# 612975; RRID: AB_2870247
FCM: CD11b BUV661, rat monoclonal anti-mouse (clone M1/70), dilution 1:640	BD Biosciences	Cat# 612977; RRID: AB_2870249
FCM: CD11b BUV395, rat monoclonal anti-mouse(clone M1/70), dilution 1:400	BD Biosciences	Cat# 563553; RRID: AB_2738276
FCM: Ly-6C BV711, rat monoclonal anti-mouse (clone HK1.4), dilution 1:800	Biolegend	Cat# 128037; RRID: AB_2562630
FCM: Ly-6G BV421, rat monoclonal anti-mouse (clone 1A8), dilution 1:300	Biolegend	Cat# 127628; RRID: AB_2562567
FCM: F4/80 BV421, rat monoclonal anti-mouse (clone BM8), dilution 1:200	Biolegend	Cat# 123131; RRID: AB_2563102
FCM: CD49d BV421, rat monoclonal anti-mouse (clone R1-2), dilution 1:160	BD Biosciences	Cat# 564397; RRID: AB_2738789
FCM: CD206 APC, rat monoclonal anti-mouse (clone C068C3), dilution 1:50	Biolegend	Cat# 141708; RRID: AB_10900231
FCM: CD206 BV421, rat monoclonal anti-mouse (clone C068C3), dilution 1:200	Biolegend	Cat# 141717; RRID: AB_2562232
FCM: CD206 BV650, rat monoclonal anti-mouse (clone C068C3), dilution 1:200	Biolegend	Cat# 141723; RRID: AB_2562445
FCM: I-A/I-E (MHCII) AF700, rat monoclonal anti-mouse (clone M5/114.15.2), dilution 1:800	Biolegend	Cat# 107621; RRID: AB_493726
IF: GFP, chicken polyclonal to GFP, dilution 1:1000	Abcam	Cat# ab13970; RRID: AB_300798
IF: mCherry, chicken polyclonal to mCherry, dilution 1:200	Abcam	Cat# ab183628; RRID: AB_2650480
IF: MMR/CD206, chicken polyclonal anti-mouse, dilution 1:300	R&D systems	Cat# AF2535; RRID: AB_2063012
IF: CD31, rat monoclonal anti-mouse (clone MEC 13.3), dilution 1:300	BD Biosciences	Cat# 550274; RRID: AB_393571
IF: CD68, rat monoclonal anti-mouse (clone FA-11), dilution 1:200	Bio-Rad	Cat# MCA1957; RRID: AB_322219
IF: anti-chicken IgG (H + L) AF488, dilution 1:500	Jackson ImmunoResearch	Cat# 703-545-155; RRID: AB_2340375
IF: anti-rabbit IgG (H + L) AF555, dilution 1:500	Thermo Fisher Scientific	Cat# A-31572; RRID: AB_162543
IF: anti-rat IgG (H + L) AF647, dilution 1:500	Abcam	Cat# ab150155; RRID: AB_2813835
IF: anti-goat IgG (H + L) AF755, dilution 1:500	Thermo Fisher Scientific	Cat# SA5-10091; RRID: AB_2556671

**Chemicals, peptides, and recombinant proteins**		

DMEM-F12 (1:1), GlutaMAX	Gibco, Thermo Fisher	Cat# 31331028
HBSS	Gibco, Thermo Fisher	Cat# 14175-095
Penicillin/Streptomycin	Gibco, Thermo Fisher	Cat# 15140122
Fetal Bovine Serum (FBS)	Gibco, Thermo Fisher	Cat# 10270-106
Trypsin-EDTA (0.05%), phenol red	Gibco, Thermo Fisher	Cat# 25300054
PBS	Gibco, Thermo Fisher	Cat# 20012027
Tissue-Tek® O.C.T. Compound	Tissue-Tek, Sakura Finetek	Cat# 4583
Triton X-100	Applied Chemicals	Cat# A4975
Fluorescence Mounting Medium	Dako, Agilent	Cat# S302380
Bovine Serum Albumin	Jackson ImmunoResearch	Cat# 001-000-162
UltraPure™ 0.5M EDTA	Invitrogen	Cat# 15575020
Normal donkey serum	SigmaAldrich	Cat# S30-M
Pentobarbital	CHUV Hospital, Lausanne, Switzerland	N/A
Gadobutrol (Gadovist)	Bayer	N/A
Attane™ Isoflurane	Attane	N/A

**Critical commercial assays**		

Zombie NIR Fixable Viability Kit	BioLegend	Cat# 423106
4′, 6- Diamidino-2-Phenylindole, Dihydrochloride (DAPI)	Life Technologies	Cat# D1306
Brain Tumor Dissociation Kit (P)	Miltenyi	Cat# 130-095-942
Myelin Removal Beads II kit	Miltenyi	Cat# 130-096-433
Translucent High Density PET Membrane, 0.4 μm pores	Falcon	Cat# 353493

**Experimental models: Cell lines**		

MMTV-PyMT (murine mammary tumor virus; Polyoma middle T antigen); PyMT-BrM3	Croci et al.^1^ Prof. J.A. Joyce	Available upon request
LLC (Lewis lung carcinoma); LLC-BrM3	This study, Prof. J.A. Joyce	Available upon request
B78 mCherry/OVA	Garcia-Martin et al.^2^ Prof. R. Lyck	Available upon request
J774A.1	ATCC	Available upon request

**Experimental models: Organisms/strains**		

C57BL/6J mice	The Jackson Laboratory	RRID: IMSR_JAX:000664

**Recombinant DNA**		

Plasmid: GFP	I. Malanchi	N/A
Plasmid: sLP-mCherry	Ximbio	Cat# 155083
pCMV delta R8.2	Addgene	Cat# 12263
pCMV-VSV-G	Addgene	Cat# 8454
Plasmid: MSCV-IRES-mCherry	W. Held	N/A

**Software and algorithms**		

FlowJo, version 10.5.3	Tree Star	https://www.flowjo.com/
GraphPad Prism v10.0.3	GraphPad Software	https://www.graphpad.com/scientific-software/prism/
ZEN software	Zeiss	https://www.zeiss.com/microscopy/en/products/software.html
QuPath version 0.4.2	Bankhead et al.^3^	https://qupath.github.io/
IDEAS® V6.2	Cytek Biosciences	https://cytekbio.com/pages/imagestream#tab-options

**Other**		

gentleMACS Octo Dissociator	Miltenyi	Cat# 130-095-937
gentleMACS C Tubes	Miltenyi	Cat# 130-096-334
LS Columns	Miltenyi	Cat# 130-042-401
Fortessa flow cytometer	BD Bioscience	N/A
Axio Scan.Z1 slide scanner	Zeiss	N/A
Imagestream X MKII	Cytek Biosciences	N/A
Tissue-Tek Cryomold	Sakura	Cat# 4557
Hydrophobic pen	Daido Sangyo, Plano	Cat# 22304
Coverslip	ThermoFisher	Cat# BB02400500A113FST0
Microscope slide	Fisherbrand	Cat# 12-550-15
Vetbond tissue adhesive	3M	Cat# 1469SB

### Resource availability

#### Lead contact

Further information and requests for resources and reagents should be directed to, and will be fulfilled by, the lead contact Prof. Johanna Joyce (johanna.joyce@unil.ch).

#### Materials availability

PyMT-BrM3 GFP/sLP–mCherry, PyMT-BrM3 GFP/mCherry, LLC-BrM3, LLC-BrM3 GFP/sLP–mCherry were generated for this study and they are available upon request to the lead contact.

#### Data and code availability

• Data reported in this paper will be shared by the lead contact upon request.
• This paper does not report original code.
• Any additional information required to reanalyze the data reported in this paper is available from the lead contact upon request.

### Experimental model and study participant details

#### Cell lines

The PyMT-BrM3 cell line was generated as previously described.^24^ Briefly, the brain-homing capacity of a cell line first isolated from a metastatic lymph node from an MMTV-PyMT mouse was enriched for via three rounds of *in vivo* selection and *in vitro* expansion. LLC-BrM3 cells were generated for this study using a similar strategy to increase the brain-homing capacity, starting from parental LLC cells (purchased from the ATCC). The J774A.1 cell line was purchased from the ATCC. The B78 mCherry/OVA cell line was kindly provided by Prof. Ruth Lyck, Theodor Kocher Institute, University of Bern, Switzerland.^28^ All cell lines were maintained in DMEM-F12 media (Gibco, ThermoFisher, cat. no. 31331028) supplemented with 10% FBS (Gibco, ThermoFisher, cat. no. 10270-106), 1% penicillin/streptomycin (P/S, Gibco, ThermoFisher, cat. no. 15140122). To generate PyMT-BrM3 GFP/sLP–mCherry cells, the original unlabeled PyMT-BrM3 cell line was transduced with GFP and sLP–mCherry plasmids as previously described.^6^^,^^7^ Briefly, cancer cells were transduced using a second-generation lentivirus coding for GFP (provided by Prof. I. Malanchi), consequently transduced with a lentivirus coding for sLP-mCherry (provided by Prof. I. Malanchi), and finally FACS-sorted for enriching GFP+ mCherry+ cells. To generate PyMT-BrM3 GFP/mCherry cells, the original unlabeled PyMT-BrM3 cell line was transduced using a lentivirus coding for GFP (provided by Prof. I. Malanchi) and subsequently with a mouse stem cell virus (MSCV) coding for the mCherry construct (provided by Prof. W. Held), and lacking the sLP portion (but otherwise identical to the sLP–mCherry construct).

#### Animals

Wild-type C57BL/6J mice were bred within the University of Lausanne animal facilities, and all animal studies were first approved by the Institutional Animal Care and Use Committees of the University of Lausanne and Canton Vaud, Switzerland under licenses VD3314 and VD3688.

#### Brain metastasis in vivo models

To generate breast-BrM, 6- to 10-week-old females were injected in the left cardiac ventricle with 1x10^5^ PyMT-BrM3 cells resuspended in 100 μL HBSS (Gibco, ThermoFisher, cat. no. 14175-095) under isoflurane anesthesia (O_2_ + 2% isoflurane). To generate a single parenchymal lung-BrM lesion, LLC-BrM3 cells were injected intracranially. 25x10^3^ LLC-BrM3 cells resuspended in 0.5 μL HBSS were inoculated intracranially in 6- to 8-week-old males. To detect BrM, mice were imaged weekly by 3T MRI (Bruker), following intraperitoneal (i.p.) injection with 150 μL Gadobutrol (Gadovist, 1 mmol mL^−1^, Bayer). Mice were sacrificed and tissue was collected at the humane endpoint of the experiment.

### Method details

#### Bone marrow-derived macrophage generation

Bone marrow was harvested from femurs and tibias of female C57BL/6J mice. Cells were filtered using a 70 μm strainer (Greiner, cat. no. 352350). Cells were centrifuged and resuspended at 0.75x10^6^ cells/ml in RPMI 1640 media (Gibco, ThermoFisher, cat. no. 61870010) supplemented with 10% FBS +1% P/S + CSF-1 (M-CSF) 50 ng/mL (Miltenyi, cat. no. 130-101-700). Then, 1.5x10^6^ cells were plated in a 6-well plate. Two days after cell plating, 500 μL of fresh complete media with CSF-1 was added to each well. On day 5 and 7 after cell plating, media was replaced with fresh complete media with CSF-1. On day 7, BMDMs were activated by adding either LPS O111B4 20 ng/mL (Merck, cat. no. L2630) or IL-4 20 ng/mL (R&D systems, cat. no. 404-ML) to the complete media with CSF-1. On day 8, BMDMs were harvested in PBS with EDTA 10 nM (Thermo Fisher Scientific, cat. no. 15575020) by pipetting on ice after 10 min, and co-cultured with cancer cells as described below.

#### Co-culture experiments

For the *in vitro* assays involving J774A.1 as the macrophage source, 7x10^5^ PyMT-BrM3 GFP/sLP–mCherry, PyMT-BrM3 GFP/mCherry, or B78 mCherry/OVA cells were co-cultured with 9x10^4^ J774A.1 cells directly or in the presence of a high density, translucent PET membrane with 0.4 μm pores (Falcon, cat. no. 353493) in a 6-well plate in DMEM-F12 media +10%FBS +1% P/S. After 72 h, cells were collected using 0.05% Trypsin (Gibco, ThermoFisher, cat. no. 25300054) and then stained with antibodies listed in Table S1 for flow cytometry and imaging flow cytometry analyses, and with antibodies listed in Table S2 for cytospin IF analyses. For the *in vitro* assays involving BMDMs as the macrophage source, 7x10^5^ PyMT-BrM3 GFP/sLP–mCherry, PyMT-BrM3 GFP/mCherry, or unlabeled PyMT-BrM3 cells were stained with Cell Tracker deep red (Invitrogen, cat. no. C34565) for 45 min at 37°C, diluted 1/1000 in PBS, followed by two washes in complete media. Cancer cells were then co-cultured with 9x10^4^ activated or control BMDMs directly in a 6-well plate in DMEM-F12 media +10%FBS +1% P/S. After 72 h, cells were collected in PBS with EDTA 10 nM (Thermo Fisher Scientific, cat. no. 15575020) by pipetting on ice after 10 min, and then stained with antibodies listed in Table S1 for flow cytometry analyses, and with antibodies listed in Table S2 for cytospin IF analyses.

#### Flow cytometry

Mice were euthanized by terminal anesthesia using pentobarbital (Lausanne University Hospital, CHUV), followed by transcardial perfusion with PBS (Gibco, ThermoFisher, cat. no. 20012027). Tissue was digested using a Brain Tumor Dissociation Kit (P) (Miltenyi, cat. no. 130-095-942) following the manufacturer’s protocol. The cell suspension was filtered using a 100 μm filter and washed with FACS buffer (PBS, 0.5% BSA (Jackson ImmunoResearch, cat. no. 001-000-162), 2mM EDTA (Thermo Fischer Scientific, cat. no. 15575020)). Myelin was removed using Myelin Removal Beads II (Miltenyi, cat. no. 130-096-433) following the manufacturer’s protocol. To exclude dead cells from analysis, cells were stained with Near Far-Red Zombie (BioLegend, cat. no. 423106) in PBS. The single-cell suspension was stained with antibodies listed in Table S1 For the co-culture experiment, the single-cell suspension was directly stained with antibodies listed in Table S1 All antibodies were purchased from BD Bioscience and BioLegend. Flow cytometry data were acquired using an LSR Fortessa (BD Bioscience), and data were analyzed with FlowJo Software v10.5.3 (Tree Star).

#### Imaging flow cytometry

Cells were stained as reported in the flow cytometry section (antibodies listed in Table S1) and up to 20,000 total events were acquired using a using a dual camera ImageStream X MKII (Cytek Biosciences) and a 60× magnification lens. Single stained controls were acquired to generate a compensation matrix using the software IDEAS V6.2 (Amnis). Focus cells were selected and single cells were identified based on the area and aspect ratio of the brightfield channel. CD45^+^ cells were gated on negative live/dead marker and the Bright Detail Similarity R3 of CD206 and mCherry was determined by the software IDEAS V6.2 as log transformed Pearson’s correlation coefficient of the localized bright spots with a radius of three pixels or less within the brightfield area in the two channels.

#### Immunofluorescence staining of co-culture experiments and analysis

Cells in the direct co-cultures were collected using 0.05% Trypsin (Gibco, ThermoFisher, cat. no. 25300054). Cells were washed and resuspended at 1x10^6^ cells/ml in PBS +40% FBS. 100 μm of the cell solution were loaded into a cytofunnel (Fisher scientific, cat. no. 11911788) with a slide attached, and cells were spun at 700 rpm for 3 min at room temperature. Then, the cytofunnels were removed and the slides were air-dried for 1 h at room temperature. Cells were fixed on the slide with periodate-lysine-paraformaldehyde (PLP) buffer for 15 min at room temperature. Slides were washed twice in PBS and cells were then permeabilized with 0.1% Triton X-100 (PanReac AppliChem, cat. No. A4975) in PBS for 10 min at room temperature and blocked in 10% donkey serum (EMD Millipore, Merck, cat. No. S30-M) in PBS for 1 h at room temperature, followed by incubation with primary antibodies (listed in Table S2) in PBS with 5% donkey serum overnight at 4 °C. Secondary staining was performed in PBS with 5% donkey serum with antibodies (listed in Table S2) for 1 h at room temperature. Finally, slides were mounted with mounting media (Dako, cat. No. S302380) and a coverslip (Menzel-Gläser, Thermo Scientific, cat. No. 631–0973). Stained tissue sections were imaged with an Axio Scan.Z1 slide scanner (Zeiss) with a Colibri 7 LED light source (Zeiss) using a Plan-Apochromat ×20/0.8 DIC M27 objective (Zeiss). 2D images were analyzed using QuPath v0.4.2.

#### Immunofluorescence staining of tissue sections and analysis

Mice were euthanized by terminal anesthesia using pentobarbital (Lausanne University Hospital, CHUV), followed by transcardial perfusion with PBS and PLP. Perfused organs were further fixed overnight in PLP buffer at 4°C and then transferred to 30% sucrose overnight at 4°C. Tissues were embedded in OCT (Tissue-Tek, Sakura Finetek, cat. No. 4583) and 10-μm cryostat tissue sections of frozen tissue were used for subsequent analyses. Tissues were permeabilized, stained, and slides acquired as indicated in the section above. 2D images were analyzed using QuPath v0.4.2. Cells in the brain parenchyma were stratified based on the shorter distance from the tumoral area.

### Statistical analysis

Prism 10 was used to perform statistical analysis and graphically plot all data. Data were tested for normal distribution with the Shapiro-Wilk test. Parametric data were analyzed by a paired two-tailed Student’s t test. Non-parametric data were analyzed by a paired two-tailed Student’s t test with Wilcoxon correction. *p* < 0.05 was considered statistically significant. Each specific statistical test used is reported for each experiment in the figure legends.

## Supplemental information

Document S1. Figures S1‒S4 and Tables S1 and S2
