# Supplementary material for: Investigation of a fluorescent reporter microenvironment niche labeling strategy in experimental brain metastasis
Source: iScience. 2024 Jun 15;27(7):110284. doi: 10.1016/j.isci.2024.110284 (PMC11261144; doi:10.1016/j.isci.2024.110284)
Supplement: Document S1. Figures S1‒S4 and Tables S1 and S2 [file mmc1.pdf]

iScience, Volume *27*

Supplemental information

Investigation of a fluorescent reporter microenvironment niche labeling strategy in experimental brain metastasis

Matteo Massara, Bastien Dolfi, Vladimir Wischnewski, Emma Nolan, Werner Held, Ilaria Malanchi, and Johanna A. Joyce

Figure S1

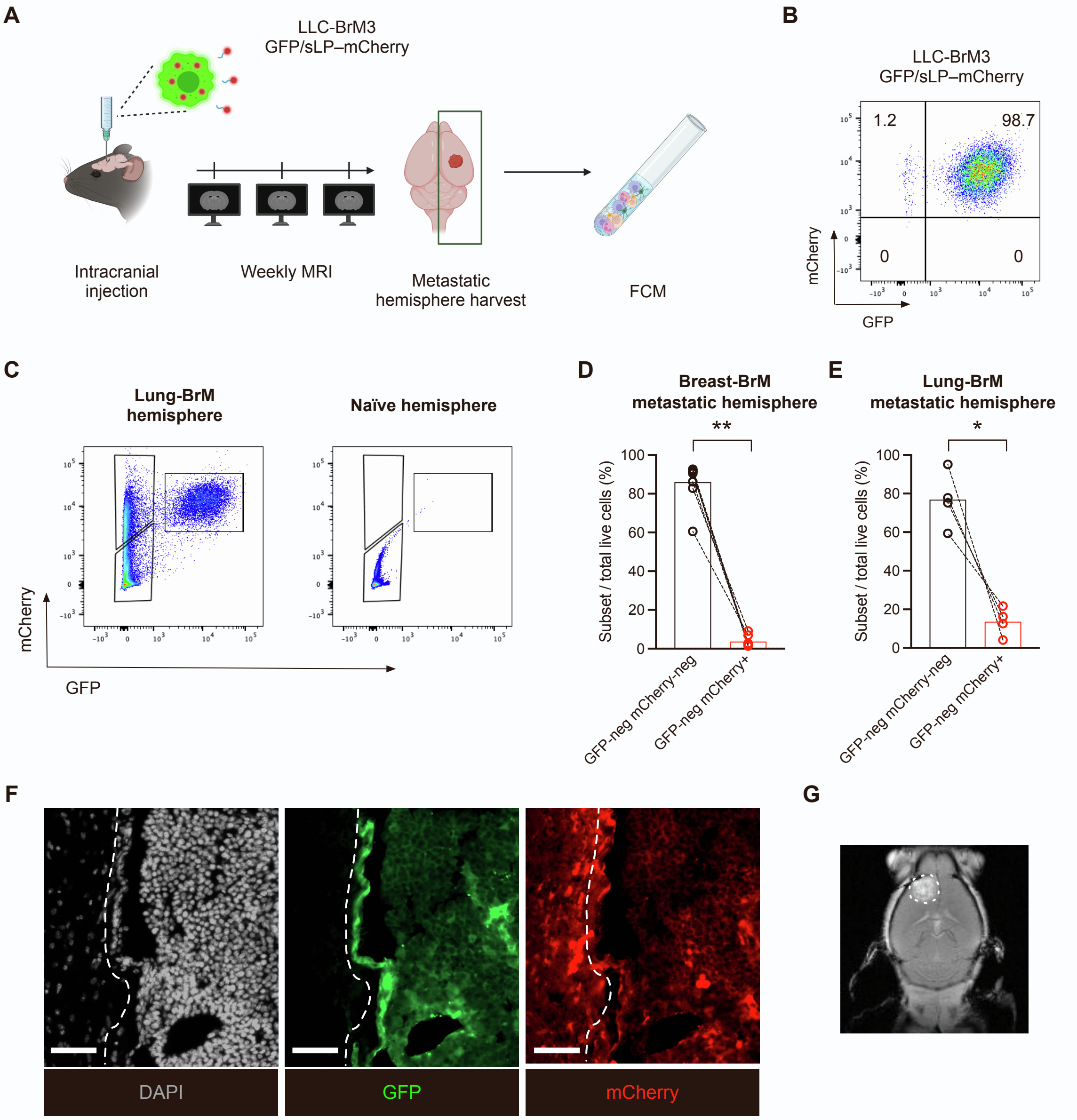
A
LLC-BrM3
GFP/sLP–mCherry
Intracranial
injection
Weekly MRI
Metastatic
hemisphere harvest
FCM
B
LLC-BrM3
GFP/sLP–mCherry
1.2
98.7
0
0
mCherry
GFP
C
Lung-BrM
hemisphere
Naïve hemisphere
mCherry
GFP
D
Breast-BrM
metastatic hemisphere
**
Subset / total live cells (%)
100
80
60
40
20
0
GFP-neg mCherry-neg
GFP-neg mCherry+
E
Lung-BrM
metastatic hemisphere
*
Subset / total live cells (%)
100
80
60
40
20
0
GFP-neg mCherry-neg
GFP-neg mCherry+
F
DAPI
GFP
mCherry
G

Figure S1. The mCherry-niche labeling system in established lung-BrM and breast-BrM, related to Figure 1

(A) Schematic for the experimental investigation of the metastatic niche in lung-BrM. Briefly, immunocompetent mice were injected intracranially with GFP/sLP–mCherry-transduced LLC-BrM3 cells and monitored by weekly MRI to assess BrM outgrowth. At the experimental endpoint, the brain hemispheres where the BrM was detected by MRI were collected by performing a sagittal cut and processed for FCM analysis. **(B)** Representative FCM plot of GFP/sLP–mCherry LLC-BrM3 cells cultured *in vitro*. **(C)** Representative FCM plots of single-cell suspensions from the labeled LLC-BrM3 lung-to-brain metastatic hemisphere (left) and the naïve uninjected brain tissue (right) used as a negative reference for mCherry expression in the FCM analyses. **(D, E)** Relative quantification of both mCherry-neg and mCherry+ cells in the (D) breast-BrM and (E) lung-BrM metastatic hemispheres. Dashed lines indicate paired samples. Metastatic hemispheres from n = 8 mice for the breast-BrM model and n = 4 for the lung-BrM model. **(F)** Representative single channels of IF staining for the representative image shown in Fig. 1D, panel i, with the GFP+ mCherry+ cancer cells at the right side of the image. Scale bar 50 μm. **(G)** Representative MRI of the brain sample shown in Fig. 1D for IF analysis. The BrM is encircled by a white dotted line. Statistical analysis in (D) was performed using paired t-test with Wilcoxon correction, and in (E) using paired t-test. *, $p < 0.05$; **, $p < 0.01$.

Figure S2

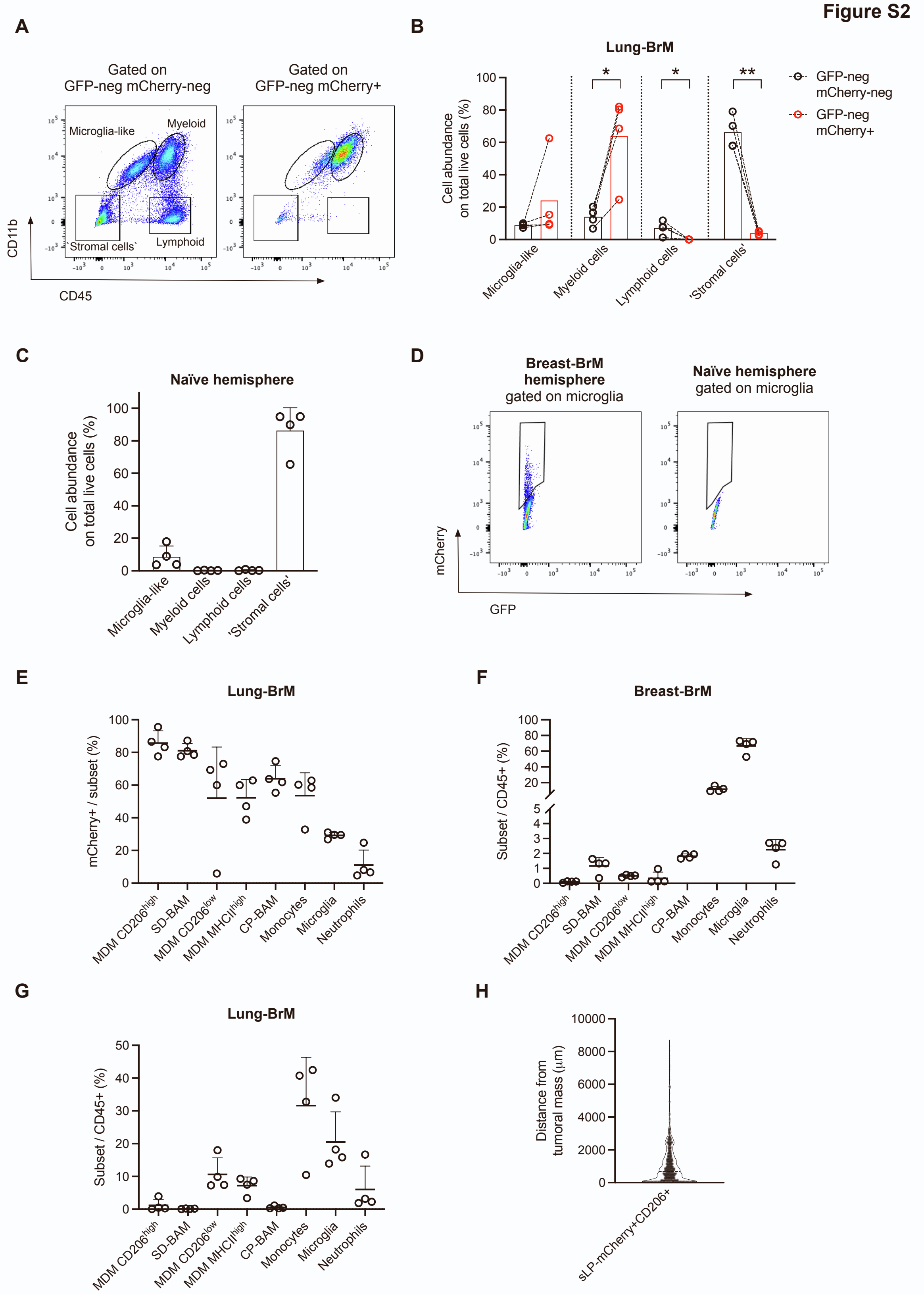
A
Gated on GFP-neg mCherry-neg
Gated on GFP-neg mCherry+
Microglia-like
Myeloid
'Stromal cells'
Lymphoid
CD11b
CD45
B
Lung-BrM
Cell abundance on total live cells (%)
GFP-neg mCherry-neg
GFP-neg mCherry+
Microglia-like
Myeloid cells
Lymphoid cells
'Stromal cells'
C
Naïve hemisphere
D
Breast-BrM hemisphere gated on microglia
Naïve hemisphere gated on microglia
mCherry
GFP
E
Lung-BrM
mCherry+ / subset (%)
MDM CD206high
SD-BAM
MDM CD206low
MDM MHCIIhigh
CP-BAM
Monocytes
Microglia
Neutrophils
F
Breast-BrM
Subset / CD45+ (%)
G
Lung-BrM
Subset / CD45+ (%)
H
Distance from tumoral mass (µm)
sLP-mCherry+CD206+

Figure S2. Tumor-associated macrophages are the most abundant cell population in the mCherry+ fraction in established lung-BrM, related to Figure 2

(A) Representative flow cytometry plots of the GFP-neg mCherry-neg and GFP-neg mCherry+ fractions in the lung-BrM model. Microglia-like cells were defined as $CD45^{low}$ $CD11b^{high}$, myeloid cells as $CD45^{high}$ $CD11b^{high}$, lymphoid cells as $CD45^{high}$ CD11b-neg, and 'stromal cells' as CD45-neg CD11b-neg. **(B)** Relative quantification of cell populations in the GFP-neg mCherry-neg and in the GFP-neg mCherry+ fractions in the lung-BrM model. Dashed lines indicate paired samples. Metastatic hemispheres from n = 4 mice. **(C)** Relative quantification of the cell populations indicated in healthy uninjected brains, naïve hemispheres from n = 4 mice. **(D)** Representative FCM plots of mCherry and GFP expression of microglia from breast-BrM or naïve non-tumor containing brain hemispheres. Naïve brain tissue was used as a negative reference for mCherry expression for the FCM analysis. **(E)** Quantification of relative mCherry uptake by the different cell populations in the lung-BrM model. Metastatic hemispheres from n = 4 mice. **(F, G)** Quantification of the different myeloid subsets as a proportion of the total immune (CD45+) fraction in the metastatic hemisphere in the (F) breast- and (G) lung-BrM models. n = 4 for both breast- and lung-BrM metastasis. **(H)** Distance distribution of sLP-mCherry+CD206+ cells from the tumoral mass, as assessed by IF staining of whole tissue sections. Each dot represents an individual cell. n = 3672 sLP-mCherry+CD206+ cells. Pooled data from the metastatic brains of n = 5 mice. Statistical analysis in (B) was performed using paired t-test. Data are represented as mean ± SD. *, $p < 0.05$; **, $p < 0.01$.

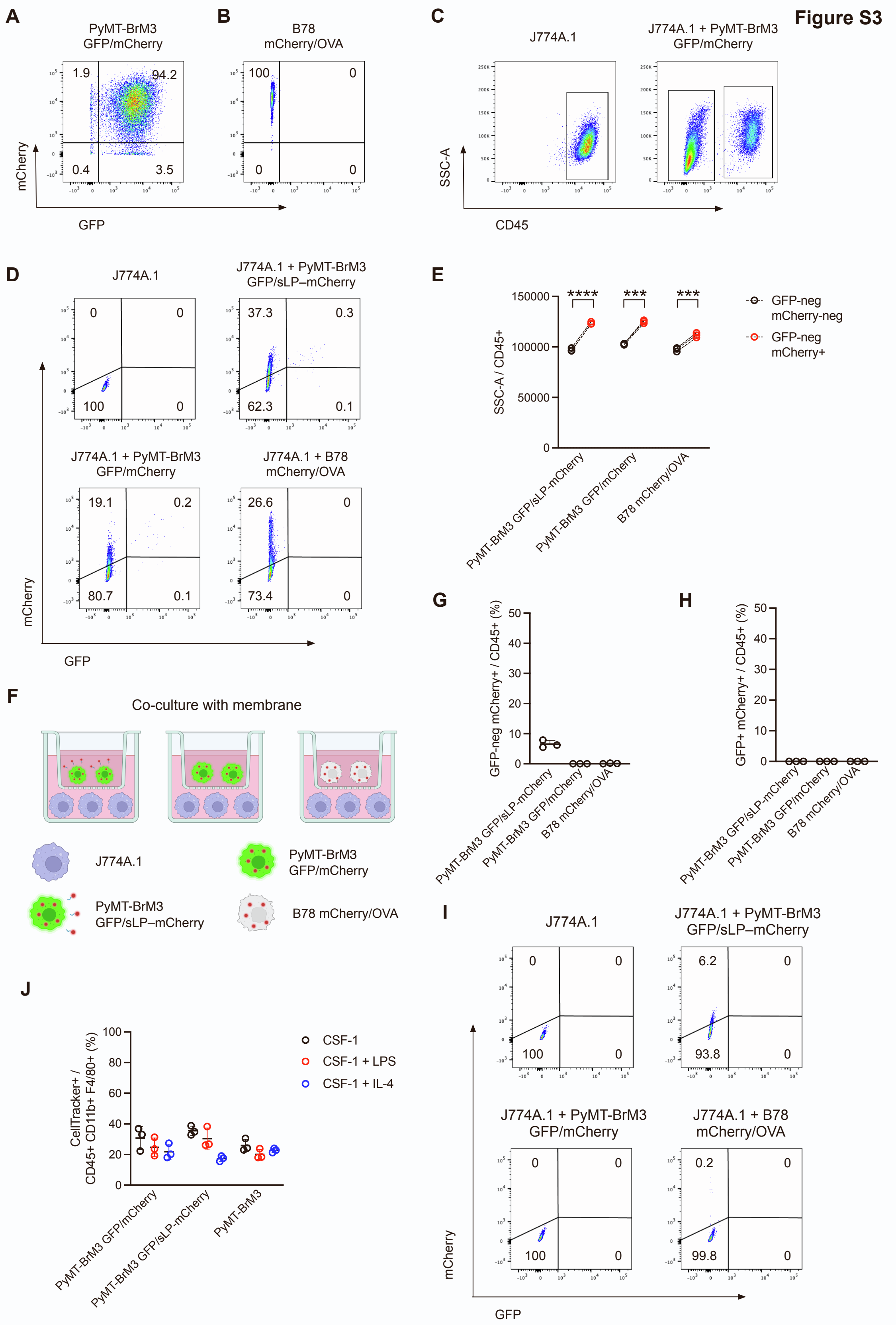
Figure S3
A
PyMT-BrM3 GFP/mCherry
1.9
94.2
0.4
3.5
mCherry
GFP
B
B78 mCherry/OVA
100
0
0
0
C
J774A.1
J774A.1 + PyMT-BrM3 GFP/mCherry
SSC-A
CD45
D
J774A.1
J774A.1 + PyMT-BrM3 GFP/sLP–mCherry
J774A.1 + PyMT-BrM3 GFP/mCherry
J774A.1 + B78 mCherry/OVA
mCherry
GFP
E
SSC-A / CD45+
GFP-neg mCherry-neg
GFP-neg mCherry+
PyMT-BrM3 GFP/sLP-mCherry
PyMT-BrM3 GFP/mCherry
B78 mCherry/OVA
F
Co-culture with membrane
J774A.1
PyMT-BrM3 GFP/mCherry
PyMT-BrM3 GFP/sLP–mCherry
B78 mCherry/OVA
G
GFP-neg mCherry+ / CD45+ (%)
H
GFP+ mCherry+ / CD45+ (%)
I
J774A.1
J774A.1 + PyMT-BrM3 GFP/sLP–mCherry
J774A.1 + PyMT-BrM3 GFP/mCherry
J774A.1 + B78 mCherry/OVA
J
CellTracker+ / CD45+ CD11b+ F4/80+ (%)
CSF-1
CSF-1 + LPS
CSF-1 + IL-4
PyMT-BrM3 GFP/mCherry
PyMT-BrM3 GFP/sLP-mCherry
PyMT-BrM3

Figure S3. J774A.1 macrophages retain mCherry *in vitro*, related to Figure 3

(A-B) Representative FCM plots of (A) PyMT-BrM3 GFP/mCherry (without the sLP portion) and (B) B78 mCherry/OVA. **(C)** Representative FCM plots of J771A.1 macrophages alone or co-cultured with PyMT-BrM3 GFP/mCherry cancer cells. **(D)** Representative FCM plots of J771A.1 macrophages detected as CD45+ events, alone or co-cultured with different transduced cancer cell lines; PyMT-BrM3 GFP/sLP–mCherry, PyMT-BrM3 GFP/mCherry, or B78 mCherry/OVA. **(E)** SSC-A values of J774A.1 detected as CD45+ cells, either GFP-neg mCherry-neg or GFP-neg mCherry+. Dashed lines indicate paired samples. **(F)** Schematic of the experimental design and the cell line used for co-cultures analyses, in the presence of a membrane insert to physically separate the two indicated cell types. **(G, H)** Relative quantification of (G) GFP-neg mCherry+ and (H) GFP+ mCherry+ expression by J774A.1 macrophages, detected as CD45+ cells by FCM. **(I)** Representative FCM plots of J771A.1 cells alone or co-cultured with different transduced cancer cell lines in the presence of the membrane insert; PyMT-BrM3 GFP/sLP–mCherry, PyMT-BrM3 GFP/mCherry, or B78 mCherry/OVA. **(J)** Relative quantification of Cell Tracker uptake by BMDMs, detected as CD45+ CD11b+ F4/80+ cells by FCM. In panels (E, G, H) n = 3 samples per group, experiment repeated 3 independent times. In panel (J) n = 3 samples per group, experiment repeated 2 independent times, one representative experiment is shown. Statistical analysis in (E) was performed using paired t-test. Data are represented as mean ± SD. ***, $p < 0.001$; ****, $p < 0.0001$.

Figure S4

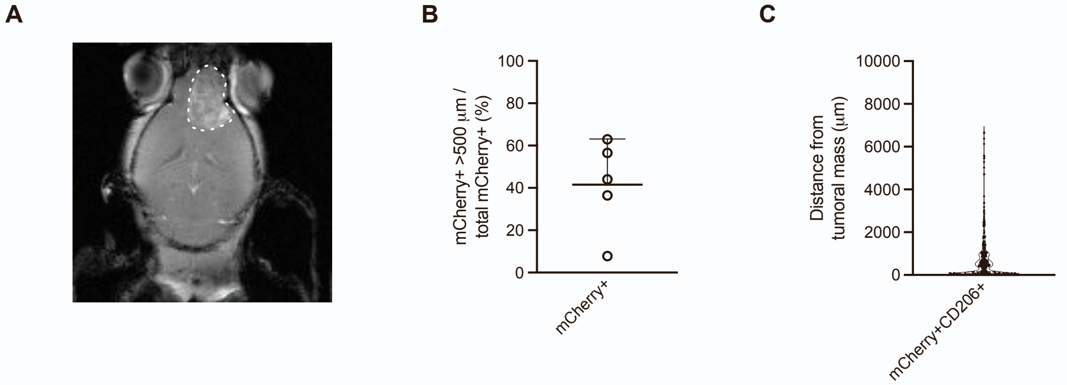
A
B
C
100
80
60
40
20
0
mCherry+ >500 μm / total mCherry+ (%)
mCherry+
10000
8000
6000
4000
2000
0
Distance from tumoral mass (μm)
mCherry+CD206+

Figure S4. mCherry labels CD206+ cells in breast-BrM, related to Figure 4

(A) Representative MRI of the brain sample shown in Fig. 4B for IF analysis. The BrM lesion is encircled by a white dotted line. **(B)** Ratio of the number of mCherry+ DAPI+ cells detected at a distance from the BrM lesion (> 500 μm) over the total mCherry+ DAPI+ fraction. n = 5 metastatic hemispheres. **(C)** Distance distribution of mCherry+CD206+ cells from the tumoral mass, as assessed by IF staining of whole tissue sections. Each dot represents an individual cell. n = 513 mCherry+CD206+ cells. Pooled data from the metastatic brains of n = 5 mice.

Supplementary Tables

Table S1. Antibody list for flow cytometry (FCM) analysis, related to STAR Methods

Antibody	Fluorophore	Clone	Manufacturer	Catalog number	Dilution
CD45	AF700	30-F11	Biolegend	103128	1:200
CD45	BUV661	30-F11	BD Bioscience	612975	1:500
CD11b	BUV661	M1/70	BD Bioscience	612977	1:640
CD11b	BUV395	M1/70	BD Bioscience	563553	1:400
Ly-6C	BV711	HK1.4	Biolegend	128037	1:800
Ly-6G	BV421	1A8	Biolegend	127628	1:300
F4/80	BV421	BM8	Biolegend	123131	1:200
CD49d	BV789	R1-2	BD Bioscience	564397	1:160
CD206	APC	C068C2	Biolegend	141708	1:50
CD206	BV421	C068C2	Biolegend	141717	1:200
CD206	BV650	C068C2	Biolegend	141723	1:200
MHCII	AF700	M5/114.15.2	Biolegend	107621	1:800
Zombie NIR	Near infrared		Biolegend	423106	1:800

Table S2. Antibody list for immunofluorescence (IF) staining, related to STAR Methods

Antibody	Species	Clone	Manufacturer	Catalog number	Dilution
GFP	Chicken	Polyclonal	Abcam	ab13970	1:1000
mCherry	Rabbit	Polyclonal	Abcam	ab183628	1:200
CD206	Goat	Polyclonal	R&D systems	AF2535	1:300
CD31	Rat	MEC 13.3	BD Bioscience	550274	1:300
CD68	Rat	FA-11	Bio-Rad	MCA1957	1:200
Anti-chicken IgG (H+L) AF 488	Donkey	Polyclonal	Jackson ImmunoResearch	703-545-155	1:500
Anti-rabbit IgG (H+L) AF 555	Donkey	Polyclonal	Thermo Fisher Scientific	A-31572	1:500
Anti-rat IgG (H+L) AF 647	Donkey	Polyclonal	Abcam	ab150155	1:500
Anti-goat IgG (H+L) AF 755	Donkey	Polyclonal	Thermo Fisher Scientific	SA5-10091	1:500
Anti-rat IgG (H+L) AF 755	Donkey	Polyclonal	Thermo Fisher Scientific	SA5-10031	1:500
DAPI			Life Technologies	D1306	1:2000
